# Deep learning signature of brain [^18^F]FDG PET associated with cognitive outcome of rapid eye movement sleep behavior disorder

**DOI:** 10.1038/s41598-022-23347-x

**Published:** 2022-11-10

**Authors:** Hyun Gee Ryoo, Jung-Ick Byun, Hongyoon Choi, Ki-Young Jung

**Affiliations:** 1 Department of Nuclear Medicine, Seoul National University Hospital, 101, Daehak-Ro, Jongno-Gu, Seoul, 03080 Republic of Korea; 2 Department of Nuclear Medicine, Seoul National University Bundang Hospital, Seongnam, Republic of Korea; 3 Department of Neurology, Kyung Hee University Hospital at Gangdong, Kyung Hee University School of Medicine, Seoul, Republic of Korea; 4 Department of Neurology, Seoul National University Hospital, 101, Daehak-Ro, Jongno-Gu, Seoul, 03080 Republic of Korea; 5 Neuroscience Research Institute, Seoul National University College of Medicine, Seoul, Korea; 6 Department of Nuclear Medicine, Seoul National University College of Medicine, Seoul, Republic of Korea

**Keywords:** Sleep disorders, Positron-emission tomography, Brain imaging, Machine learning, Predictive markers

## Abstract

An objective biomarker to predict the outcome of isolated rapid eye movement sleep behavior disorder (iRBD) is crucial for the management. This study aimed to investigate cognitive signature of brain [^18^F]FDG PET based on deep learning (DL) for evaluating patients with iRBD. Fifty iRBD patients, 19 with mild cognitive impairment (MCI) (RBD-MCI) and 31 without MCI (RBD-nonMCI), were prospectively enrolled. A DL model for the cognitive signature was trained by using Alzheimer’s Disease Neuroimaging Initiative database and transferred to baseline [^18^F]FDG PET from the iRBD cohort. The results showed that the DL-based cognitive dysfunction score was significantly higher in RBD-MCI than in RBD-nonMCI. The AUC of ROC curve for differentiating RBD-MCI from RBD-nonMCI was 0.70 (95% CI 0.56–0.82). The baseline DL-based cognitive dysfunction score was significantly higher in iRBD patients who showed a decrease in CERAD scores during 2 years than in those who did not. Brain metabolic features related to cognitive dysfunction-related regions of individual iRBD patients mainly included posterior cortical regions. This work demonstrates that the cognitive signature based on DL could be used to objectively evaluate cognitive function in iRBD. We suggest that this approach could be extended to an objective biomarker predicting cognitive decline and neurodegeneration in iRBD.

## Introduction

Isolated rapid eye movement (REM) sleep behavior disorder (iRBD) is a parasomnia characterized by the pathological lack of atonia during REM sleep^1^. The iRBD has been regarded as a preclinical disease entity with prognostic relevance as it starts from an isolated syndrome with normal cognition but eventually develops synucleinopathy including Parkinson’s disease (PD), dementia with Lewy Body (DLB), and multiple system atrophy (MSA)^2,3^. Patients with iRBD are at risk of cognitive impairment^4,5^, and about 50% of them have mild cognitive impairment (MCI)^6^. As cognitive impairments are associated with whether the patient will convert to dementia or parkinsonism first, the development of biomarkers that predict cognitive decline or MCI is gaining attention^7^.

[^18^F]fluorodeoxyglucose (FDG) PET has been used to support the differential diagnosis of dementia^8^ and parkinsonism^9,10^ and also used to predict the progression of cognitive dysfunctions in PD as well as MCI as an imaging-derived biomarker^11–15^. Because of recent advances in the deep learning (DL) technique, a cognitive signature based on DL has been suggested in multiple disease domains to predict cognitive outcome^16,17^. The DL-based model has advantages in a flexible application such as a model transfer to different disease domains^18^.

We aimed to apply a cognitive signature of [^18^F]FDG PET based on DL in patients with iRBD. The DL model trained by a relatively large dataset of Alzheimer’s disease (AD) was transferred to classify iRBD with and without MCI and predict the patient who will develop future cognitive decline or neurodegeneration.

## Methods

### Subjects

Consecutive video-polysomnography confirmed iRBD patients were prospectively enrolled between May 2017 and November 2019. RBD was diagnosed according to the International Classification of Sleep Disorders (ICSD-3) criteria^1^. Individuals with (1) neurodegenerative disease or other neurological disorders, (2) severe medical illness, or (3) moderate to severe obstructive sleep apnea (apnea–hypopnea index ≥ 20) were excluded. The demographic factors, years of education, and scores of a questionnaire evaluating RBD symptoms (Korean version of the Rapid Eye Movement Sleep Behavior Disorder Questionnaire-Hong Kong, RBDQ-KR)^34^ were reviewed for each patient. The patients were followed up annually after the initial enrollment. Conversion to synucleinopathy including PD, DLB, or MSA was based on the treating neurologist's evaluation at the time of follow-up if they met established clinical criteria for their respective diagnosis^35–37^. This study was approved by the institutional review board of Seoul National University Hospital and Kyung Hee University Hospital at Gangdong (IRB No. 1702-150-835 and 2020-05-022, respectively). Written informed consent to participate was obtained from the patients enrolled. All methods were carried out in accordance with relevant guidelines and regulations.

### Neuropsychological assessment

The Korean version of the Consortium to Establish a Registry for Alzheimer’s Disease (CERAD-K), which includes twelve cognitive subtests^38^ was used to evaluate cognitive function at baseline and 2 years after the initial enrollment. CERAD total scores (CERAD-TS) were obtained by summing scores from the individual CERAD subtests with exclusion of Mini-Mental Status Examination in the Korean Version of the CERAD Assessment Packet (MMSE-KC). The performance on each test is presented as a z-score based on age-, sex-, and education-adjusted normative data^39^. The CERAD-K was repeated at the follow-up visit and change in cognitive function was evaluated by changes in the CERAD-TS during the follow-up period.

MCI was diagnosed based on the following criteria: (1) a subjective cognitive complaint by the patient or caregiver on the structured interview, (2) an objective cognitive decline (> 1 standard deviation below the standardized mean in one or more cognitive domains), (3) preserved activities of daily living, and (4) cognitive deficits not suitably explained by medication use or other medical/psychiatric disorders. All MCI individuals had an overall clinical dementia rating (CDR) index^40^ of 0.5 as well as a CDR memory score of 0.5. The progression to MCI and overt neurodegenerative disorder were also assessed according to the follow-up visits. Patients who showed progression to overt neurodegenerative disorder less than 2 years were regarded as patients with early phenoconversion.

### [^18^F]FDG PET acquisition and processing

For the iRBD cohort, baseline [^18^F]FDG PET scans were acquired in a single institute. Images were obtained by a fully integrated simultaneous PET/magnetic resonance imaging (MRI) system with a 3-T magnet (Biograph mMR; Siemens Healthcare, Erlangen, Germany). 370 MBq of [^18^F]FDG was intravenously injected after fasting for at least 6 h and PET scanning of the brain was performed approximately 30 min later. Ultrashort echo time (UTE) sequence brain MR images were obtained to generate attenuation map. The attenuation map was obtained by an automatic 3-tissue segmentation map using UTE brain MR. [^18^F]FDG PET images were reconstructed onto 344 × 344 matrices with a 2.0 mm slice thickness using a three-dimensional iterative algorithm. The images of iRBD patients were spatially normalized to MNI space using the statistical parametric mapping software (SPM8, https://www.fil.ion.ucl.ac.uk/spm/software/spm8/). The matrix size of spatially normalized PET images was 79 × 95 × 68 and the voxel size was 2 × 2 × 2 mm^3^.

### Deep learning for cognitive dysfunction evaluation

In this study, [^18^F]FDG PET images collected from subjects recruited in Alzheimer’s Disease Neuroimaging Initiative (ADNI) were used as a training dataset for our model (https://adni.loni.usc.edu). The model was trained to discriminate [^18^F]FDG PET of AD from those of normal controls (NCs). Detailed methods and the training cohort were described in the previous study^16^. Demographic and clinical characteristics of ADNI cohort are summarized in Supplementary Table S1.

To define a cognitive signature of [^18^F]FDG PET based on DL, a convolutional neural network (CNN) model for discriminating AD from NCs was trained by ADNI datasets consisting of 637 [^18^F]FDG PET. The same CNN architecture from our previous work was used. We only modified the model to be optimized for the new version of packages including Tensorflow (version 2.0.0)^16^. Briefly, four sequential 3-dimensional (3D) convolution layers and rectified linear units (ReLU) were applied. The sizes of each convolutional filter were (5 × 5 × 5), and the stride size of 4, 2, 1, and 1 voxel were applied for the first, second, third, and fourth convolution filters respectively. After multiple convolutional filters, a global average pooling layer summarized 128 3D feature volumes into a vector for each feature map. ReLU and dropout layers were followed, and a sigmoid activation function was applied for the output layer. The details of the CNN architecture are summarized in Supplementary Table S2.

Then, the model was transferred to images of prospective cohort comprising iRBD patients with MCI (RBD-MCI) and without MCI (RBD-nonMCI) at baseline for identifying the cognitive signature of [^18^F]FDG PET. We only removed a sigmoid activation function from the transferred model for the output layer to estimate the DL-based cognitive dysfunction score of the subjects in iRBD cohort. Additionally, we employed the CAM visualization method to demonstrate the brain regions where the CNN model evaluated for cognitive dysfunction as we did in our previous study^41^. More specifically, the feature map by removing the last pooling layer showed the important area for the decision that indirectly explains how the deep CNN model predicts the outputs^42,43^. We produced CAM to visualize the area most indicative of the cognitive dysfunction of individual iRBD patients.

### Statistical analysis

Values are expressed as the mean [SD]. The accuracy of the DL-based cognitive dysfunction score was measured by the area under curve (AUC) of receiver operating characteristic (ROC) analysis. Group differences in demographic and clinical variables in iRBD cohorts were evaluated using the chi-square test and Mann–Whitney U test. Spearman’s ρ was used to test the significance of the correlation between DL-based cognitive dysfunction score and clinical value. Statistical tests were performed using a commercial software package (MedCalc version 19.5.3, MedCalc Software bvba, Ostend, Belgium), and a *P*-value lower than 0.05 was considered statistically significant.

## Results

### Patient characteristics

A total 50 of patients with iRBD were enrolled and 19 of them met the criteria for MCI at baseline. Demographics and clinical characteristics of the patients are summarized in Table 1, and the patients also participated in our previous study^19^. The education years and RBDQ-KR scores were not significantly different between RBD-MCI and RBD-nonMCI. MMSE-KC and CERAD-TS z-score of RBD-MCI were significantly lower than those of RBD-nonMCI. The mean age was older in RBD-MCI than in RBD-nonMCI. The mean duration of RBD was longer in RBD-MCI than in RBD-nonMCI.

**Table 1:** Demographics, clinical characteristics, and DL-based cognitive dysfunction scores of iRBD cohort.

	RBD-nonMCI	RBD-MCI	*P*-value
Number of subjects (2-year follow-up)	31 (12)	19 (9)	
Age (years)	64.6 (5.9)	69.6 (7.4)	0.014
**Sex, No. (%)**			
Female	11 (35.5)	10 (52.6)	n.s
Male	20 (64.5)	9 (47.4)	
RBD duration (years)	6.5 (5.1)	8.1 (3.6)	0.037
Education (years)	12.3 (3.9)	12.1 (3.8)	0.837
RBDQ-KR	38.9 (17.1)	46.0 (19.4)	0.107
MMSE-KC	28.2 (1.1)	27.3 (1.3)	0.019
CERAD-TS z-score	1.05 (0.48)	− 0.11 (0.86)	< 0.01
DL-based cognitive dysfunction score	− 1.157 (2.126)	− 0.202 (1.526)	0.018

*RBD* rapid eye movement sleep behavior disorder, *MCI* mild cognitive impairment, *RBD-nonMCI* iRBD without MCI, *RBD-MCI* iRBD with MCI, *RBDQ-KR* Korean version of rapid eye movement sleep behavior disorder questionnaire-Hong Kong, *MMSE-KC* Mini-Mental State Examination Korean version, *CERAD* Consortium to Establish a Registry for Alzheimer’s Disease, *TS* total score, *n.s* not significant.

Unless otherwise indicated, data are expressed as mean (standard deviation).

### Cognitive signature based on DL for differentiating baseline RBD-MCI from RBD-nonMCI

**Figure 1:**
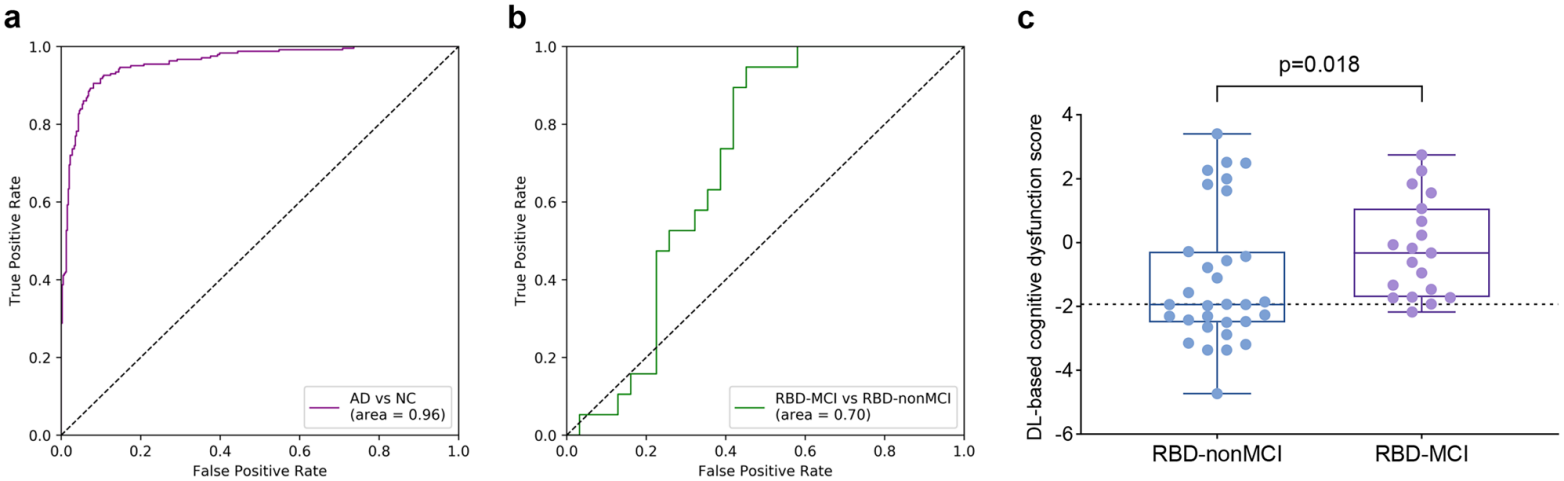
Deep learning-based cognitive signature of [^18^F]FDG PET for differentiating RBD-MCI from RBD-nonMCI. (**a**) The AUC of ROC curves for differentiating AD from NCs in ADNI cohort was 0.96 (95% CI 0.94–0.97). (**b**) The AUC of ROC curves for differentiating RBD-MCI from RBD-nonMCI was 0.70 (95% CI 0.56–0.82). (**c**) DL-based cognitive dysfunction score was significantly higher in RBD-MCI than in RBD-nonMCI (*P* = 0.018).

The model accuracy for differentiating AD from NCs was evaluated by the AUC of ROC curve, which showed 0.96 (95% CI 0.94–0.97) for the ADNI cohort (Fig. 1a). The transfer of the model was performed to evaluate [^18^F]FDG PET-based cognitive dysfunction score using DL for iRBD patients. The DL-based cognitive dysfunction score was directly applied to the task of differentiating baseline RBD-MCI from RBD-nonMCI. The AUC of ROC curves was 0.70 (95% CI 0.56–0.82) for 50 iRBD patients (Fig. 1b), and the optimal cutoff DL-based cognitive dysfunction score for the detection of iRBD with MCI was − 1.930 with a sensitivity of 94.7%, and a specificity of 54.8%. The output of the model, i.e*.,* DL-based cognitive dysfunction score, was significantly higher in RBD-MCI than in RBD-nonMCI at baseline (mean [SD], − 0.202 [1.526] vs. − 1.157 [2.126], *P* = 0.018; Fig. 1c, Table 1).

### Association between changes in cognitive function and DL-based cognitive dysfunction score

**Figure 2:**
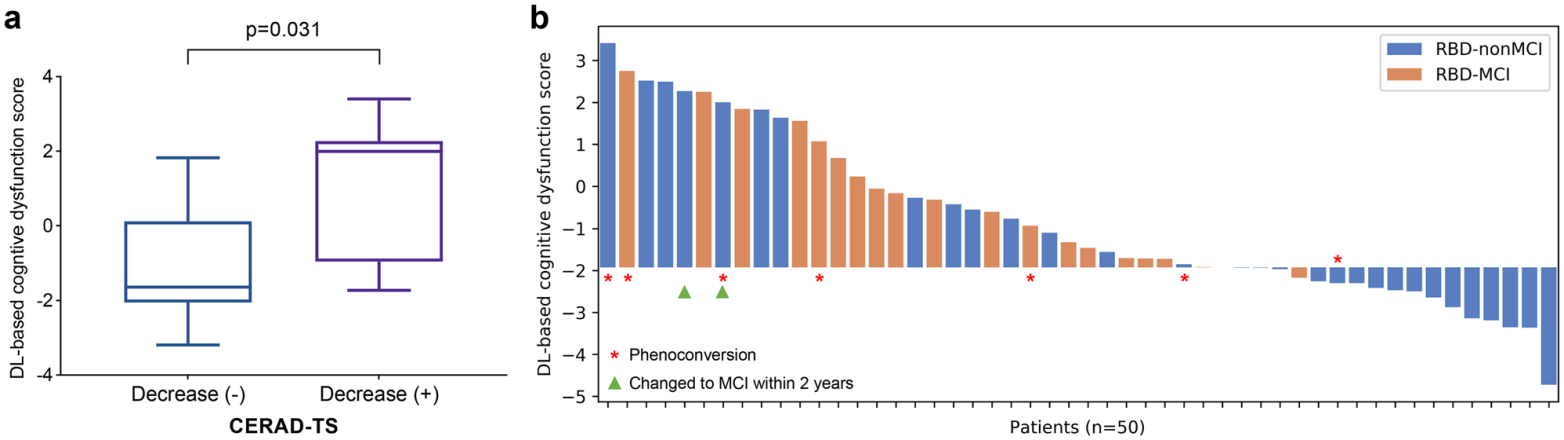
Association of longitudinal cognitive change in iRBD with DL-based cognitive dysfunction score. (**a**) DL-based cognitive dysfunction scores were higher in iRBD patients who showed a decrease in CERAD-TS on a 2-year follow-up than in those who did not. (**b**) DL-based cognitive dysfunction scores of RBD-MCI and RBD-nonMCI at baseline are visualized in the waterfall plot. The bars with asterisks or triangles indicate patients converted to overt neurodegenerative disease or MCI within 2 years.

The longitudinal change in cognitive function during 2 years was evaluated in twenty-one patients. Clinical variables including age, duration of RBD, years of education, RBDQ-KR, MMSE-KC scores, CERAD-TS z-score, and baseline DL-based cognitive dysfunction scores were similar between those who were followed up for 2 years and those who were not (Supplementary Table S3). Among 21 iRBD patients who underwent a 2-year follow-up, 7 of them showed a decrease in CERAD-TS z-score and their DL-based cognitive dysfunction score was significantly higher than those of other 14 patients who did not show a decrease in CERAD-TS z-score during 2 years (0.947 [1.989] vs. − 1.000 [1.480], *P* = 0.031; Fig. 2a). Of note, no significant correlation was observed between the DL-based cognitive dysfunction score and baseline CERAD-TS (*P* = 0.750) in these 21 patients: 6 out of 7 individual CERAD subtests scores did not show significant correlation with DL-based cognitive dysfunction score (Verbal fluency [VF], *P* = 0.964; modified Boston Naming Test [BNT],* P* = 0.192; Word List Memory [WLM],* P* = 0.327; Word List Recall [WLR],* P* = 0.782; Word List Recognition [WLRc],* P* = 0.072; Constructional Recall [CR],* P* = 0.209), but only Constructional Praxis (CP) subtest score showed significant correlation (*P* = 0.010). Among the 12 RBD-nonMCI patients at baseline who underwent a 2-year follow-up, 2 patients met the criteria for MCI during the follow-up and their DL-based cognitive dysfunction scores were not significantly different but higher than those of the other 10 patients who remained in RBD-nonMCI group (2.133 [0.191] vs. − 1.023 [2.081], *P* = 0.086; Fig. 2b). In addition, among 7 patients in RBD-nonMCI group who scored higher than the mean DL-based cognitive dysfunction score of RBD-MCI (> − 0.202), 3 out of 4 patients (75%) who followed up for 2 years converted to MCI and/or overt neurodegenerative disease.

Seven patients converted to synucleinopathy (5 PD, 1 DLB, 1 MSA) within 2 years. The DL-based cognitive dysfunction scores of these 7 iRBD patients who converted to synucleinopathy were 0.586 [2.292], which was not significantly different from those of 16 iRBD patients who did not convert to synucleinopathy within 2 years (− 0.661 [1.684], *P* = 0.256). However, when the patients were classified according to the optimal cutoff value of DL-based cognitive dysfunction score (− 1.930), 6 out of 7 patients (85.7%) with phenoconversion scored above the cutoff value (Table 2).

**Table 2:** Classification of iRBD patients with phenoconversion to overt neurodegenerative disease depending on the baseline diagnosis of MCI and optimal cutoff of DL-based cognitive dysfunction score based on [^18^F]FDG PET.

	RBD-nonMCI		RBD-MCI	
	Non-converter	Converter	Non-converter	Converter
**DL-based cognitive dysfunction score**				
Above cutoff	4	3^a^	7	3^b^
Below cutoff	5	1^c^	0	0

*RBD* rapid eye movement sleep behavior disorder, *MCI* mild cognitive impairment, *RBD-MCI* iRBD with MCI, *RBD-nonMCI* iRBD without MCI.

^a^2 PD and 1 MSA-C.

^b^2 PD and 1 DLB.

^c^1 PD.

**Figure 3:**
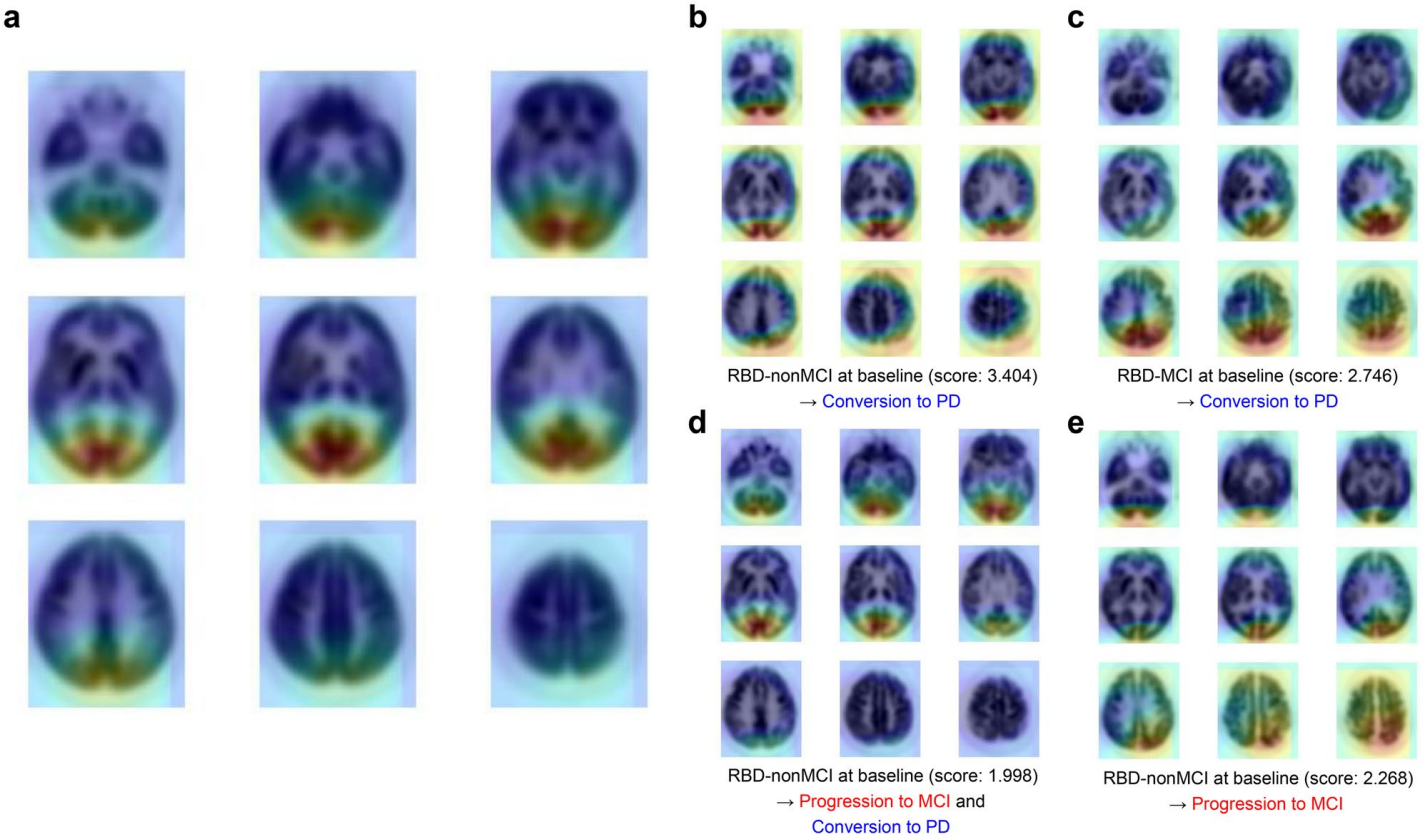
Cognitive dysfunction-related region mapping. (**a**) The averaged CAM of iRBD patients with a higher DL-based cognitive dysfunction score than the optimal cutoff value (− 1.930). Cognitive dysfunction-related regions mainly included posterior cortical regions. (**b**-**e**) Representative CAM of individuals who (**b**-**d**) converted to overt neurodegenerative disease or (**d**, **e**) progressed to MCI. The number in parentheses is DL-based cognitive dysfunction score of individuals.

In addition, the regions related to cognitive dysfunction were represented by a class activation map (CAM). In our study, brain metabolic features related to the cognitive dysfunction-related regions of individuals were partly different from each other, but the regions mainly included posterior cortical regions: occipital, posterior parietal, and temporal areas (Fig. 3). Among these posterior cortical regions, the occipital cortical area mostly showed higher weight, i.e., more impacts on the prediction of cognitive dysfunction, compared to other regions.

## Discussion

In this study, we evaluated an objective cognitive signature using [^18^F]FDG PET to associate with cognition in iRBD. The DL model showed good performance in discriminating iRBD patients with MCI from those without MCI. In addition, the DL-based cognitive dysfunction score showed a trend of correlation with longitudinal changes in cognitive function.

Our results suggested that the DL-based cognitive dysfunction score could stratify brain metabolism patterns related to cognitive dysfunction in iRBD patients as a preclinical neurodegenerative disorder. In this study, our deep CNN model trained by [^18^F]FDG PET patterns of cognitive dysfunction in AD compared with NCs was transferred to [^18^F]FDG PET of iRBD cohort to estimate individuals’ DL-based cognitive dysfunction score. Notably, the transfer of the model was directly applied to iRBD cohorts for the task of differentiating iRBD patients with MCI as our hypothesis was the existence of common metabolic patterns related to cognitive decline in AD and iRBD. This score showed the feasibility of not only classifying baseline RBD-MCI and RBD-nonMCI patients but also predictive value in future cognitive decline. A previous study showed the DL-based cognitive dysfunction score was higher in MCI converters than in non-converters of MCI patients in the ADNI cohort. In addition, the score was higher in Parkinson’s disease patients with dementia than in those without dementia^16^. According to these results, the DL-based cognitive dysfunction score may be an objective biomarker derived from [^18^F]FDG PET to assess cognitive dysfunction in various neurodegenerative disorders including iRBD as a preclinical neurodegenerative disorder.

According to our results of the model transfer, metabolic patterns related to cognitive function in iRBD may share that of cognitive disorders particularly including AD, which implies a partly common pathophysiologic process between the two. Cholinergic dysfunction is crucial pathophysiology of cognitive decline in PD as well as iRBD patients. A transcranial magnetic stimulation study suggested that widespread central cholinergic disruption may be associated with cognitive dysfunction in iRBD^20^. A [^11^C]donepezil PET study showed more widely reduced cortical acetylcholinesterase activity in iRBD patients with cognitive impairment^21^. Moreover, an amyloid PET study showed that 17.4% of patients with iRBD show positive cerebral amyloid deposit^22^. In addition, recent amyloid PET studies in patients with DLB and PD dementia showed increased amyloid deposit that was associated with cognitive dysfunction, and the amyloid deposition in DLB patients was associated with striatal dopaminergic depletion^23,24^. Altogether, these findings reflect co-existing AD pathology in iRBD for cognitive decline and neurodegeneration. Therefore, it is not surprising that the DL-based cognitive dysfunction score trained by AD patients could be transferred to the cohort of iRBD.

The regions related to cognitive dysfunction were represented by CAM, which has been used for visualizing the location of pattern identified by CNN models. Accordingly, brain metabolic features related to the cognitive dysfunction-related regions of individual iRBD patients mainly included posterior cortical regions. Notably, the pattern recognized by the CAM did not simply reflect hypometabolism but showed the location of abnormal patterns weighted on the CNN model. Thus, it could be different from the regions with hypometabolism or hypoperfusion in previous studies. Nevertheless, posterior areas have been commonly reported to be associated with cognitive function iRBD. RBD patients with MCI showed waking EEG slowing in the posterior cortical regions^25^, and reduced brain perfusion in the posterior cortical regions on SPECT^26^. Only RBD patients with MCI showed posterior cortical hypoperfusion, mainly in parietal, temporal, and particularly occipital areas compared with controls and RBD without MCI^26^. Topographical analysis of cortical cholinergic denervation was greater in the occipital cortex in patients with iRBD^21^, and network-based statistics also showed reduced cortico-cortical functional connectivity strength in the posterior regions associated with mental processing speed^27^. A recent study of voxel-wise group comparisons for FDG PET data revealed that the cognitive impairment in patients with iRBD was related to functional and metabolic changes in posterior brain regions, particularly occipital and parietal areas, and the hypometabolism in these brain regions was a predictor of phenoconversion^28^. Overall, these findings suggest that RBD patients who have an abnormality in posterior cortical regions might be at an increased risk for cognitive impairment, and eventually phenoconversion. Even though the pattern was similar in patients with high DL-based cognitive dysfunction scores, individualized spatial patterns may provide the functional correlates of brain metabolism. As a future study, predicting types of neurodegenerative disorders as well as outcomes of patients according to the spatial pattern of cognitive dysfunction-related regions suggested by the CNN model can be investigated. Also, it should be noted that the cognitive dysfunction-related region revealed by CAM in RBD-MCI was different from patterns of hypometabolism in MCI converting to AD: RBD-MCI mainly included posterior cortical regions, whereas the MCI converting to AD mainly includes temporo-parietal cortices and posterior cingulate cortex/precuneus^29,30^. Since MCI is a heterogeneous syndrome resulting from AD as well as non-AD and non-neurodegenerative conditions^31–33^, our DL-based approach may have clinical implications for the accurate diagnosis of the etiology of MCI.

The DL-based cognitive dysfunction score of iRBD patients at baseline was higher in iRBD patients who showed a future cognitive decline, represented by the changes of CERAD total scores. Even though the limited number of patients who were followed up for 2 years, our results suggested iRBD patients who showed decreased CERAD score might be predicted by the baseline DL-based cognitive dysfunction score. Furthermore, six iRBD patients with DL-based cognitive dysfunction scores above the cutoff value showed overt neurodegenerative disorders (6/17), while one iRBD patient with DL-based cognitive dysfunction scores below the cutoff value showed overt neurodegenerative disorders within 2 years (1/6). As a prospective study with relatively small samples and limited follow-up period, prediction of progression to the synucleinopathies, as well as future cognitive decline, should be investigated with a larger cohort and long-term follow-up to establish a predictive DL-based biomarker.

In this study, the AUC of ROC curves for differentiating RBD-MCI from RBD-nonMCI was relatively low compared to the classification models directly trained using labeled data which resulted from the intrinsic properties of iRBD. There is an intrinsic limitation in completely distinguishing two conditions, iRBD with or without cognitive decline, by brain imaging alone due to the relatively mild cognitive abnormality of iRBD patients compared to the AD cohort, and a lot of clinical overlap between RBD-MCI and RBD-nonMCI in terms of disease severity. In addition, PET images for training the model were spatial normalized using the bounding matrix which included the whole cerebrum and basal ganglia, considering the 3D CNN model size. These factors as well as model architectures could be further modified and our study has room for further performance improvement through optimization. Considering the main aim of this study is to prove the concept of DL-based cognitive signature applying to iRBD patients, an optimized model with more data may improve the performance of predicting cognitive function.

Some limitations should be noted. First, this study was a single-center study with a small number of patients. Second, a limited number of longitudinal clinical data was available due to the relatively short-term follow-up period compared to the long-term changes in clinical features of iRBD patients. A larger longitudinal study in patients with iRBD may be warranted. Fine-tuning of the CNN model could be performed to improve the accuracy of the model when more [^18^F]FDG PET and clinical data in iRBD are available in a larger longitudinal study. In addition, because of the small number of patients in iRBD cohort, DL-based cognitive signatures were not evaluated with other covariates. In terms of clinical variables that could affect the cognitive decline in iRBD patients, a cognitive signature from the integrated model that includes demographic features such as age and gender as input will be a more powerful model. Also, because of the absence of amyloid biomarkers, we could not compare amyloid deposits in iRBD patients. Additional studies with biomarkers with amyloid pathology could clarify co-existing AD pathology in iRBD for cognitive decline and neurodegeneration.

To conclude, the cognitive signature of brain [^18^F]FDG PET based on DL could be used to objectively evaluate cognitive function in iRBD. This may improve the ability to stratify patients with cognitive impairment and could identify the patients at higher risk of cognitive decline. We suggest that this approach may be extended to a quantitative imaging biomarker to provide the degree of neurodegeneration in iRBD in terms of the cognitive domain, leading to early detection and disease-modifying therapy.

## Supplementary Information

Supplementary Tables.

## Data Availability

The data used in this study are not publicly available. The data may be made available from the corresponding authors upon reasonable request subject to permission and approval from the corresponding organizations and institutional review boards. Code used for model training and evaluation is available from the corresponding author on reasonable request.
