# Supplementary material for: Deep learning signature of brain [18F]FDG PET associated with cognitive outcome of rapid eye movement sleep behavior disorder
Source: Sci Rep. 2022 Nov 10;12:19259. doi: 10.1038/s41598-022-23347-x (PMC9649732; doi:10.1038/s41598-022-23347-x)
Supplement: Supplementary file 1 — Supplementary Tables. [file 41598_2022_23347_MOESM1_ESM.docx]

**Supplementary Table S1.** Demographics and baseline clinical characteristics of ADNI cohort

|  | **AD (N = 243)** | **NC (N = 394)** |
| --- | --- | --- |
| Age, mean (SD), y | 75.0 (7.8) | 73.7 (5.9) |
| Sex, No. (%) |  |  |
| Female | 100 (41.2) | 199 (50.5) |
| Male | 143 (58.8) | 195 (49.5) |
| MMSE, mean (SD) | 23.2 (2.1) | 29.0 (1.2) |

Abbreviation: AD, Alzheimer’s disease; NC, normal control

**Supplementary Table S2.** CNN architecture of FDG PET-based cognitive dysfunction evaluation model

| **Layer type** | **Output size** | **Kernel size** | **Stride** | **Activation** |
| --- | --- | --- | --- | --- |
| Conv3D | 20 $\times$ 24 $\times$ 17, 32 | 5 $\times$ 5 $\times$ 5 | 4 | ReLU |
| Conv3D | 10 $\times$ 12 $\times$ 9, 32 | 5 $\times$ 5 $\times$ 5 | 2 | ReLU |
| Conv3D | 10 $\times$ 12 $\times$ 9, 64 | 5 $\times$ 5 $\times$ 5 | 1 | ReLU |
| Conv3D | 10 $\times$ 12 $\times$ 9, 128 | 5 $\times$ 5 $\times$ 5 | 1 | ReLU |
| Global Average Pooling | 128 |  |  |  |
| Dense | 128 |  |  | ReLU |
| Dense | 1 |  |  | Sigmoid |

**Supplementary Table S3.** Demographics, clinical characteristics, and DL-based cognitive dysfunction scores between iRBD patients who were followed up for 2 years and who were not

|  | **Not followed up** | **Followed up** | ***P*-value** |
| --- | --- | --- | --- |
| Number of subjects (%) | 29 (58.0) | 21 (42.0) |  |
| Age, y | 66.0 (6.0) | 67.2 (8.1) | 0.836 |
| Sex, No. (%) |  |  |  |
| Female | 16 (55.2) | 5 (23.8) | 0.028 |
| Male | 13 (44.8) | 16 (76.2) |  |
| RBD duration, y | 6.2 (3.0) | 8.2 (6.1) | 0.223 |
| Education, y | 12.0 (4.1) | 12.4 (3.4) | 0.754 |
| RBDQ-KR | 43.9 (17.4) | 38.4 (19.1) | 0.387 |
| MMSE-KC | 28.0 (1.3) | 27.8 (1.3) | 0.665 |
| CERAD-TS z-score | 0.67 (0.84) | 0.53 (0.90) | 0.582 |
| DL-based cognitive dysfunction score | -1.115 (1.993) | -0.351 (1.869) | 0.128 |

Abbreviation: RBD, rapid eye movement sleep behavior disorder; MCI, mild cognitive impairment; iRBD-nonMCI, RBD without MCI; RBD-MCI, iRBD with MCI; RBDQ-KR, Korean version of rapid eye movement sleep behavior disorder questionnaire-Hong Kong; MMSE-KC, Mini-Mental State Examination Korean version; CERAD, Consortium to Establish a Registry for Alzheimer’s Disease; TS, total score; n.s, not significant

Unless otherwise indicated, data are expressed as mean (standard deviation)
